# Effect of doxycycline and *Lactobacillus* probiotics on mRNA expression of ABCC2 in small intestines of chickens

**Published:** 2016

**Authors:** A. Milanova, I. Pavlova, V. Yordanova, S. Danova

**Affiliations:** 1 Department of Pharmacology, Physiology of Animals and Physiological Chemistry, Faculty of Veterinary Medicine, Trakia University, 6000 Stara Zagora, Bulgaria;; 2 BSc in Molecular Biology, Molecular Diagnostics Unit, Hospital for Active Treatment “Dr. Atanas Dafovski”, Kurdjali, Bulgaria;; 3 The Stephan Angeloff Institute of Microbiology, Bulgarian Academy of Sciences (BAS), 26, Akad. G. Bontchev, Sofia, Bulgaria

**Keywords:** ABC transporters, Doxycycline, *Lactobacillus* probiotics, Poultry

## Abstract

Probiotics and antibiotics are widely used in poultry and may alter drug bioavailability by affecting the expression of intestinal ATP-binding cassette (ABC) efflux transporters. Therefore the aim of the present investigation was to evaluate the effect of *Lactobacilli* probiotics, administered alone or in combination with doxycycline, on the expression of ABCB1 (gene, encoding P-glycoprotein), ABCC2 (gene, encoding multidrug resistance protein 2, MRP2) and ABCG2 (gene, encoding breast cancer resistance protein) mRNAs in chicken using RT-PCR. Duc one-day-old chicks (n=24) were divided equally in four groups: untreated control, probiotics supplemented group, probiotics plus doxycycline treated chickens and antibiotic administered group. Expression of ABCC2 mRNA was affected by doxycycline or by combination of *Lactobacillus plantarum*, *L. brevis* and *L. bulgaricus* and the antibiotic in the intestines. These results can be used as a basis for further functional studies to prove the beneficial effect on limitation of the absorption of toxins and improvement of efflux of endogenous substances and xenobiotics when the combination of doxycycline and *Lactobacillus* spp. probiotics are administered to poultry.

## Introduction

 ATP-binding cassette (ABC) efflux transporters are part of the gastro-intestinal barrier and extrude drugs, xenobiotics and metabolites (Schrickx and Fink-Gremmels, 2008 ▶), ABCB1, ABCC2 and ABCG2 mRNA are observed at high levels in small intestine and liver of chickens (Haritova, 2006 ▶; Su et al., 2014 ▶).

 Composition of feed and inflammation can modulate the expression and subsequently the function of ABC transporters. Gastro-intestinal disorders, provoked by pathogenic *Escherichia coli* can alter the expression and function of ABC efflux transporters in chickens which lead to changes in drug pharmacokinetics (Haritova, 2006 ▶; Guo et al., 2014 ▶). Apart from their control with antibiotics, probiotic supplementation of chicken feed is successfully used to reduce clinical cases of disease and to improve immune response of poultry (Kabir, 2009 ▶). There are data showing that *Lactobacilli* probiotics modulate the expression and function of MRP2 and of P-glycoprotein in the gastro-intestinal tract (Stojančević et al., 2014).

 Therefore, current study was designed to evaluate the effect of probiotics *Lactobacillus brevis*, *L. plantarum* and *L. bulgaricus*, and doxycycline on the expression of ABCB1, ABCC2 and ABCG2 mRNA in Duc broiler chickens.

## Materials and Methods

**Drug**

 Medicated water with doxycycline hyclate (200 mg/4 L water, Doxy-200 ws. Interchemie, Venray, Holland) was prepared *ex tempore* in the morning between 7.30 and 8.00 h and in the afternoon between 16 and 17 h.

**Probiotic strains**

 Probiotic strains *L. brevis* 51, *L. plantarum* 11, and *L. bulgaricus* 13, isolated from traditional dairy products, were used (Microbial Collection, Laboratory of Genetics of Probiotic Bacteria, Institute of Microbiology, BAS). They were characterized as candidate probiotics according to the *in vitro* criteria of WHO (Tropcheva et al., 2013 ▶). *Lactobacillus* strains were selected according to their possibilities to survive at the concentrations of doxycycline in the gastro-intestinal tract of poultry. They were cultured in skim milk (Humana, Holdorf, Germany), lyophilized and stored at -20°C. The lyophilized product of *L. brevis* was 1.6 × 10^6^ CFU/mg product, *L. plantarum* 1.06 × 10^6^ CFU/mg, and *L. bulgaricus* 0.25 × 10^3^ CFU/mg.

 They were administered via feed by daily supplemen-tation at a dose of 1 g/kg feed from each lyophilized strain.

**Animals and experimental design**

 One-day-old Duc broiler chickens from both sexes (n=24, “Bovans Bulgaria”, Chirpan, Bulgaria) were randomly divided into four groups and received feed and water *ad libitum*. The first group (n=6) was not treated and served as a control. The second group (n=6) received probiotics 5 days after hatching via the feed for 15 days. The third group (n=6) was treated with probiotics as described above in combination with doxycycline. The antibiotic was administered via drinking water at a dose rate of 10 mg/kg. The treatment started 15 days after hatching and lasted 5 days. The fourth group (n=6) was treated with doxycycline via drinking water at a dose rate of 10 mg/kg for 5 days, 15 days after hatching.

 The chickens were euthanized and tissue samples of duodenum, jejunum and liver were quickly removed, snap-frozen in liquid nitrogen and stored at -70°C until analysis. The samples were collected 20 days after hatching. Clinical examination confirmed the absence of any signs of diseases.

 The experiments were approved by the Ethical Commission for Animal Experiments at Trakia University, Stara Zagora (Protocol No. 65/18.10.2013).

**Real-time PCR analysis**

 Total RNA was isolated using TRItidy G (Genaxxon Bioscience GmbH, Germany). The quality and quantity of total RNA was determined by ultraviolet absorbance at 260 and 280 nm, and the samples were stored at -70°C. First-strand cDNA was synthesized using the First Strand cDNA Synthesis Kit (Fermentas Life Science, Thermo Scientific, USA) on Quanta Biotech QB-96 (Quanta Biotech Ltd., Surrey, UK). Chicken specific primers (Table 1) ABCB1, ABCC2 and ABCG2, glyceraldehyde 3-phosphate dehydrogenase (GAPDH) and hexose-6-phosphate dehydrogenase (H6PD) were obtained from Sigma (Sigma-Aldrich, UK). RT-PCR was performed with iTaq Universal SybrGreen Supermix (Bio-Rad, Hercules, CA, USA) in a StepOnePlus™ Real-Time PCR System and by StepOne^TM^ Software, v 2.1 (Applied Biosystems, Thermo Fisher Scientific, Paisley, UK). Following an initial hot-start for 3 min, each reaction went through a PCR cycle with a denaturation step at 95°C for 20 s, an annealing step specific for each set of primers for 30 s and an elongation step at 72°C for 30 s. After 35 cycles a melting curve was obtained by increasing the temperature with 0.5°C every 10 s from 65°C to 95°C. The analyses were done in triplicate. Gene expression data were presented using delta Ct analysis and the algorithms outlined by Vandesompele *et al*. (2009) and the geNorm manual (https://genorm.cmgg. be/). Efficiency of each reaction was computed with LinRegPCR 7.0 Software (Heart Failure Research Center, Amsterdam, The Netherlands).

**Statistical analysis**

 Data are presented as mean±SD. Statistical analysis was performed with ANOVA test followed by Bonferroni’s multiple comparison test. Statistically significant differences were considered at P<0.05.

## Results

**Fig. 1:**
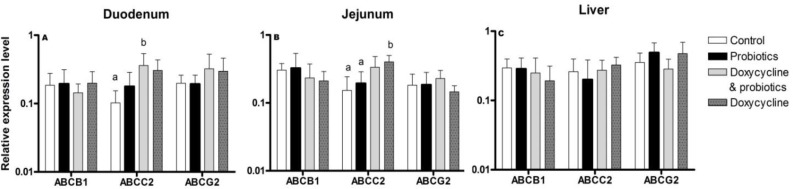
Relative expression levels (Mean±SD) of ABCB1, ABCC2, and ABCG2 mRNA in the duodenum (A), jejunum (B), and liver (C) of untreated Duc broilers (n=6), *Lactobacillus* probiotics supplemented (n=6), doxycycline and *Lactobacillus* probiotics treated (n=6), and doxycycline administered chickens (n=6). Doxycycline was administered orally via drinking water at a dose rate of 10 mg/kg. Different letters present statistically significant differences at level of P<0.05

 Supplementation of the feed with probiotics did not provoke significant changes in ABCB1, ABCC2, and ABCG2 mRNA levels (Figs. 1A, B and C). ABCB1 mRNA was insignificantly down-regulated after doxy-cycline treatment in comparison to groups supplemented with probiotics or to untreated controls. ABCC2 mRNA was significantly up-regulated in the duodenum in the group of chickens treated with doxycycline in combination with probiotics when it was compared to the control group (Fig. 1A). ABCC2 mRNA was significant-ly up-regulated after doxycycline treatment in the jejunum (Fig. 1B). ABCG2 mRNA was not altered in the studied tissues.

**Table 1:** Chicken specific primers used in the study

Gene	NCBI accession number	Forward primer 5´3´	Reverse primer 5´3´	T_a_ (°C)
ABCB1	NM_204894	GCTGTTGTATTTCCTGCTATGG	ACAAACAAGTGGGCTGCTG	58
ABCC2	XM_421698	CTGCAGCAAAATGAGAGGACAATG	CAGAAGCGCAGAGAAGAAGACCAC	63
ABCG2	XM_004942107.1	CCTACTTCCTGGCCTTGATGT	TCGGCCTGCTATAGCTTGAAATC	62
H6PD	XM_425746.4	GAGAACCAGCACTTCTTAGAC	GGGTTCAGCAACTCCACTG	64
GAPDH	NM_204305	GTGTGCCAACCCCCAATGTCTCT	GCAGCAGCCTTCACTACCCTCT	65

ABCB1: Gene encoding ATP-binding cassette subfamily B member 1, P-glycoprotein; ABCC2: Gene, encoding ABC subfamily C member 2, multidrug resistance-associated protein 2; ABCG2: Gene, encoding ABC subfamily G member 2, breast cancer resistance protein; H6PD: Hexose-6-phosphate dehydrogenase; GAPDH: Glyceraldehyde 3-phosphate dehydrogenase; NCBI: The National Centre for Biotechnology Information; Ta: Optimal annealing temperature

## Discussion

 Beneficial effect of supplementation of chicken feed with probiotics on intestinal microbiota, feed intake and digestion, and on the immune system was reviewed by Kabir (2009) ▶. Data about significant influence of wide spectrum antibiotics such as doxycycline on intestinal microbiota exist (Yin et al., 2015). Moreover, impact of probiotics and antibiotics on transporter proteins in mammals were described but published literature about poultry is scarce (Pan and Yu, 2014 ▶; Stojančević et al., 2014 ▶).

 The expression of ABCB1, ABCC2 mRNAs in the liver, duodenum and jejunum in Duc chickens was similar to the results in Ross 308 broilers (Guo et al., 2014). ABCB1 mRNA levels can be affected by the age of poultry and can differ in the health and during progress of inflammation (Guo et al., 2014 ▶). Administra-tion of *Lactobacillus* spp. with or without doxycycline did not cause any significant changes of ABCB1 mRNA which can be mentioned as properties of probiotics to keep balance in the gastro-intestinal tract not only by regulation of microbiota, but also by effect on pathogenic bacteria. Ability of probiotic *L. ramnosus* to keep gastro-intestinal integrity by restoration of ABCB1 mRNA levels was confirmed in mouse with hepatic steatosis (Wang et al., 2012 ▶). Doxycycline treatment of Duc chickens did not change ABCB1 mRNA in contrast to fluoroquinolones enrofloxacin and danofloxacin which up-regulated its levels in healthy turkeys and *E. coli* infected chickens (Haritova, 2006 ▶). These findings were supported by further functional studies and increased protein expression in *E. coli* O2 infected and enrofloxacin treated broilers (Guo et al., 2014 ▶). The presented data demonstrate that the various antibiotics changed the expression of this efflux transporter protein depending on the health status. Up-regulation of ABCC2 mRNA in the duodenum of *Lactobacillus* spp. and doxycycline supplemented broilers and in the jejunum of doxycycline treated poultry, or maintenance of its levels in the liver is likely to be beneficial, as this transporter is essential for the efflux of bilirubin glucuronides, bile acids and drug-conjugates (Borst et al., 2006 ▶). A tendency toward up-regulation of ABCG2 mRNA in the duodenum after doxycycline treatment with or without probiotics and in the liver after supplementation with *Lactobacillus* probiotics are in line with the observed results in *L. ingluviei* treated mice (Angelakis et al., 2012 ▶). Impact of these changes on the absorption and excretion of either negatively or positively charged drugs, glucuronides and sulfate conjugates (Su et al., 2014 ▶) requires clarification in functional studies.

 The findings in our study are in line with absence of changes in pharmacokinetics of doxycycline observed in Duc broilers (Pavlova, 2015 ▶). These results can serve as a basis for further functional studies to prove the beneficial effect on limitation of the absorption of toxins and improvement of efflux of xenobiotics when *Lactobacillus* spp. probiotics are administered alone or in combination with doxycycline to poultry.
